# Social media for radiologists: an introduction

**DOI:** 10.1007/s13244-015-0430-0

**Published:** 2015-09-22

**Authors:** Erik R. Ranschaert, P. M. A. van Ooijen, Simon Lee, Osman Ratib, P. M. Parizel

**Affiliations:** Department of Medical Imaging, Jeroen Bosch Ziekenhuis, Postbus 90153, 5200 ME ‘s-Hertogenbosch, The Netherlands; Department of Radiology, University of Groningen, University Medical Center Groningen, Groningen, The Netherlands; PR & Media Department, European Society of Radiology, Neutorgasse 9, 1010 Vienna, Austria; Department of Medical Imaging and Information Sciences, University Hospital of Geneva, Geneva, Switzerland; Department of Radiology, Antwerp University Hospital & University of Antwerp, Antwerp, Belgium

**Keywords:** Social media, Radiology, Internet, Telemedicine, Social networking

## Abstract

**Abstract:**

Social media, which can be defined as dynamic and interactive online communication forums, are becoming increasingly popular, not only for the general public but also for radiologists. In addition to assisting radiologists in finding useful profession-related information and interactive educational material in all kinds of formats, they can also contribute towards improving communication with peers, clinicians, and patients. The growing use of social networking in healthcare also has an impact on the visibility and engagement of radiologists in the online virtual community. Although many radiologists are already using social media, a large number of our colleagues are still unaware of the wide spectrum of useful information and interaction available via social media and of the added value these platforms can bring to daily practice. For many, the risk of mixing professional and private data by using social media creates a feeling of insecurity, which still keeps radiologists from using them. In this overview we aim to provide information on the potential benefits, challenges, and inherent risks of social media for radiologists. We will provide a summary of the different types of social media that can be of value for radiologists, including useful tips on how to use them safely and efficiently.

***Main Messages*:**

• *Online social networking enhances communication and collaboration between peers*

• *Social media facilitate access to educational and scientific information*

• *Recommendations and guidelines from policymakers and professional organisations are needed*

• *Applications are desired for efficient and secure exchange of medical images in social media*

## Introduction

**Fig. 1:**
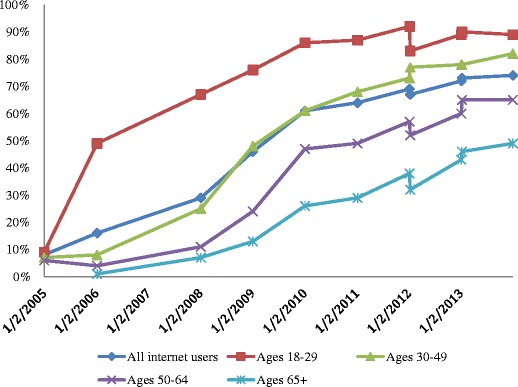
The percentage of people using social media is rising steadily. As of January 2014, 74 % of all online adults used social networking sites. For adults ages 18–29, 89 % used social networking sites; for adults ages 30–49, 82 %, and for adults ages 50–64, 65 %. Among adults ages 65+, 49 % used social networking sites. Source: Pew Research Internet Project, Pew Research Center, 2015 http://pewrsr.ch/Vhqb6S. Accessed May 2015

**Fig. 2:**
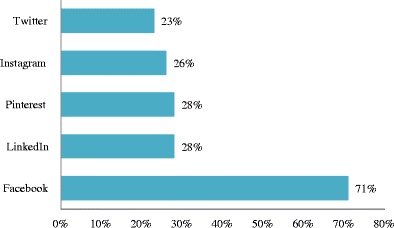
Most popular social media sites and percentage of online adults who used these websites in 2014. Source: Pew Research Internet Project, Pew Research Center, 2015 http://pewrsr.ch/Vhqb6S. Accessed May 2015

The term "social media" (SoMe) generally refers to Internet-based tools that allow individuals and organisations to communicate and to share information, ideas, personal messages, images, and other content. They have also been defined as “a group of Internet-based applications that are built on the ideological and technological foundations of Web 2.0, and that allow the creation and exchange of user-generated content” [1]. The transition in 2006 from the “read-only” Internet 1.0 to the more “wildly read-write” Internet 2.0 was a definite milestone in the history of the Internet. Its original premise was to create a network to facilitate communication and the exchange of large datasets and images, but the effect and scale of these modifications was greater than expected [2]. The rise of social networking and SoMe is one of the three major technological revolutions that have taken place in the new century, the other two being the availability of Internet and broadband connections, and the increasing integration of mobile devices and mobile connectivity into our daily lives [3]. The availability of a wide diversity of SoMe applications (apps) for mobile devices has greatly contributed to the success of SoMe, since it renders them usable for communication at any time. The ongoing two-way interactive dialogue can be live and real-time, and often people with whom we may not have had the ability to interact in real life can participate in the discussions [4]. The ever-growing popularity of SoMe among all age groups is well documented by the results of the Pew Research Internet Project (Fig. 1) [5]. As of January 2014, 74 % of all online adults were using social networking sites. The most popular platforms appear to be Facebook, LinkedIn, Pinterest, Twitter, and Instagram (Fig. 2). Given these changes, SoMe is gradually displacing the more traditional communication methods such as fax, e-mail, and even phone calls, a trend that is accelerated by the widespread use of mobile devices equipped with SoMe applications. This affects the way people communicate not only with friends and other individuals, but also with organisations, communities, hospitals and medical professionals. All these changes, together with the increasing mobility of data, will have a major impact on healthcare, and will influence the evolution of our professional activities as medical doctors and radiologists. Training and education of physicians and staff on how to deal with these new technologies is therefore essential [6]. The purpose of this article is to discuss the opportunities and challenges that SoMe provides for medicine and radiology.

## Online social networking of patients

An increasing number of patients are using the Internet to look for health information. Many patients are turning to social networking in their search for information, social support, and advice. Results from a 2014 European survey show that a majority of European citizens (59 %) are using the Internet to search for health-related information [7]. In the Eurobarometer 411 on Patient Safety and Quality of Care, it was reported that SoMe and Internet platforms were the third main source of information on healthcare quality for European patients (26 %) [8]. Patients now want their healthcare providers to use SoMe for a variety functions, including appointment reminders, diagnostic test result reporting, and health information sharing. They see it as a chance to strengthen their personal ownership of their health [4, 9]. Physicians have also begun to develop an interest in interacting with patients through social media such as Twitter and Facebook. Several studies, however, have shown that there is still considerable resistance to using this type of communication, either for personal or professional reasons [10]. Though still in its infancy, this new form of communication does have the potential to revolutionize the way physicians interact, both with their fellow healthcare workers and with patients.

## Online social networking of physicians and radiologists

The interaction of healthcare professionals on SoMe can be personal, professional, or both. According to a 2012 study, the majority (61 %) of doctors were passively scanning SoMe for medical information, whereas 46 % actively contributed to that information on a weekly basis [11]. A more recent study from the MedData Group confirmed that more than half of all physicians (56 %) used SoMe for professional purposes. The most popular platform among physicians is Facebook, which is used by 61 % of all doctors for private reasons and by only 15 % for professional purposes [12]. A 2014 Australian survey showed that most doctors (78.6 %) used SoMe only during non-working hours, which could be due to the lack of time during office hours or to the fact that they used them mostly for private purposes [13]. Other reasons for physicians to use SoMe are the opportunity to market their practice and to establish themselves as thought leaders with a high impact factor (so-called influencers) [14].

A relatively new phenomenon is the use of social media during scientific (radiological) meetings. Since 2012, the European Society of Radiology (ESR) has provided a “social media wall” during its annual meeting, the European Congress of Radiology (ECR). This medium brings together ECR-related posts and comments from various social media sources in one convenient “stream”. The messages are displayed on screens throughout the venue and can also be viewed online. In a recent article in the Journal of the American College of Radiologists (JACR), the authors concluded that the 30 % increase in the use of Twitter during the 2011 and 2012 Radiological Society of North America (RSNA) annual meetings presents an opportunity to leverage this technology to engage meeting attendees, improve scientific sessions, and increase collaboration at national radiology meetings (see Twitter and microblogging section for more information on Twitter) [7]. Nowadays, so-called TweetChats are organised at the annual scientific meetings of the American College of Radiologists (ACR), the RSNA, and the American Society of Neuroradiology (ASNR). A “TweetChat” is a pre-arranged chat or discussion on Twitter that happens by including a predefined hashtag referring to the meeting in every tweet (e.g. #ACR). In this way, all meeting-related tweets are linked in a live Twitter conversation between attending and non-attending radiologists. Several well-known radiologists including Bruce Hillman, editor-in-chief of the Journal of the American College of Radiology (JACR), and Ruth Carlos, deputy editor of the JACR, hold regular TweeChats, which they consider a great way to make connections both with colleagues within the radiology community, as well as with the greater healthcare community and patients [15]. In the ASNR 2015 TweetChat on the role of social media in scientific meetings, it was concluded that the main advantage of TweetChats is the fact that they *“…allow attendees to post comments about sessions, engage in dialogue about content, and interact with non-attendees”*. The participants also found that SoMe offered a powerful means of networking with colleagues and following multiple sessions simultaneously. They were also of the opinion that the role of SoMe would continue to increase, and that they could even turn into real-time peer reviewers of the sessions [16, 17]. In Tables 1, 2, and 3, we provide a schematic overview of the most important effects and consequences of the availability of SoMe for both patients and medical professionals (Table 1), the potential benefits of SoMe for radiologists (Table 2), and the reasons for radiologists to engage in online social networking (Table 3).

**Table 1:** Effects and consequences of the presence of social media in healthcare

- Has an impact on communication between medical professionals and patients	
- Provides possibilities to share and store medical information, including medical images	
- Offers great potential, pending further research and policymaking	
- Creates a need to train doctors for the digital era

**Table 2:** Potential benefits and opportunities of social media for radiologists

- Improvement of radiologists’ visibility among clinicians and patients	
- Increased interaction with clinicians regionally, nationally, and globally	
- Exchange and availability of relevant information and knowledge	
- Distribution and discussion of information and cases for education and research	
- Sharing and discussion of radiological images with peers and clinicians	
- Increased impact and influence in the radiological community	
- More active engagement during scientific meetings (Tweetups, Tweet Chats)	
- Augmentation of the reach of scientific publications by promotion on SoMe

**Table 3:** Reasons for radiologists to engage in social networking

1. To build, develop and maintain a network of professional contacts	
- Communicate with colleagues from local or personal network	
- Meet colleagues from around the globe	
- Collaborate with colleagues/people with common interests and experience	
2. To discover new career, research, or business opportunities	
- Find new (unpublished) opportunities	
- Establish research collaborations (virtual teams)	
- Develop business relationships, new ventures	
3. To remove barriers to improved collaboration	
- Share media in all kinds of formats	
- Discuss the latest radiology news, articles, conferences	
- Seek help or consultation from a community of experts	
- Learn from colleagues with common clinical interests/expertise	
4. To make themselves more visible to the public, a virtual “face of radiology”	
- Inform the public about radiology examinations	
- Discuss imaging-related topics with patients	
5. To access education and research	
- Subscribe to pages of radiological societies	
- Follow and participate in online discussion of cases	
- Participate in online research networks	
- Promote and discuss scientific publications	

## Privacy matters, legal issues, and guidelines

One of the major challenges of using SoMe is to ensure that, if any patient information is exchanged, it is anonymised and transmitted securely to avoid a breach of confidentiality. On the other hand, the benefits afforded by an open and accessible online platform can also threaten the privacy of the users themselves. Clinical practitioners and radiologists willing to participate in SoMe should have valid concerns about the protection of both the patients' and their own personal information when posting information or personal opinions on social networks. This concern is fueled by the fact that it is not unusual for social media users to be connected to overlapping networks of friends, family, and colleagues. One way to safeguard patient confidentiality is to give users the option of separating their personal and professional accounts, although a more practical solution is to customize the privacy settings of the platform through which individuals can protect their profile content and decide who can view it [10]. Privacy and legal concerns are driving medical doctors’ reluctance to participate more fully in SoMe. In a recent survey among Australian doctors, participants expressed their concerns about legal issues involved in online communication with patients [13]. Similar concerns have been expressed in other countries, despite the publication of guidelines by professional organisations and societies on how to avoid the pitfalls and uphold professional values when using social media. European legal rules have been established to ensure that personal data enjoy a high standard of protection; however, the current EU Data Protection Directive 95/46/EC does not adequately account for important aspects including globalization and technological developments such as social networks and cloud computing. The European Commission plans to unify data protection within the European Union (EU) with a single law, the General Data Protection Regulation (GDPR). The EU's European Council aims for adoption of a reform on the EU Data Protection Directive in 2015, and the regulation is planned to take effect after a transition period of two years [18]. More information on this topic can be found on the websites displayed in Table 4.

**Table 4:** Information on European legislation regarding data protection

Topic	Hyperlink
Reform of data protection legislation	http://bit.ly/1nN50Ah
Progress on EU data protection reform	http://bit.ly/1cSL4YF
General Data Protection Regulation	http://bit.ly/1IQ1qFC

Today, the ultimate responsibility remains with the individual professional user. According to the ACR Reference Guide in Information Technology for the Practicing Radiologist, it is “*… the responsibility of the radiologist to securely and effectively utilize mobile technology in the best interests of patient care*” [19]. Many physicians are also using open cloud storage services (e.g. Dropbox and Google Drive) and social media platforms (e.g. iMessage, Instagram and WhatsApp) to share medical and radiological images, largely because these services are free and easy to use. There are two major concerns related to the use of SoMe and open cloud services for such purposes, particularly for healthcare professionals: reliability and security [20]. Such methods of sharing medical images and sensitive data constitute a risk to the patient’s privacy, since they are designed neither for distributing nor storing protected patient-related information. Information that could identify a patient, such as birth date, address, social security number, images of a patient’s faces and/or recognizable body marks (e.g. scars, tattoos), are sensitive data that theoretically can be used for fraudulent purposes. Healthcare providers using public platforms for professional reasons cannot guarantee that no one else is able to access this information. In addition, most patients are not aware of the fact that such media containing their personal information are being used. Several organisations and hospitals have set forth policies to address these issues, e.g. by forbidding visitors and personnel to take pictures with mobile devices that have immediate access to social media. Patients should also be informed of the privacy protections put in place by their physician or physician’s practice, and should be able to provide consent to participate. The emergence of mobile technology and communication for radiologists should be embraced, under the condition that clear guidelines and proper security mechanisms are adopted to control their use in the hospital and medical working place [21]. According to Eric Topol, during the second half of this decade, we will need to pay equal attention to deep medical learning to preempt illness and to data security to protect the privacy of individuals [22]. The most important pitfalls of SoMe for radiologists are summarized in Table 5

**Table 5:** Pitfalls and dangers of social media for radiologists

- Insufficient or inappropriate legislation and policies in hospitals/practices	
- Insufficient *separation* of personal and professional data	
- Insufficient privacy settings (who can see/read what I post?)	
- Insufficient protection of patient privacy/identity	
- Lack of obvious guidelines as to what is appropriate or inappropriate to say	
- Questionable reliability of information on resources	
- Lack of review of published material	

## Practical information

SoMe platforms are used mainly for sharing information, pictures, audio, and text among a group of people who are linked by a common characteristic such as friends, family, profession, or hobbies. The purposes for which SoMe can be used by radiologists are numerous:

- Accessing news and information on radiology
- Social and professional networking (Facebook, LinkedIn)
- Scientific networking (ResearchGate, BiomedExperts)
- Blogging (WordPress, Blogger, Tumblr)
- Microblogging (e.g. Twitter)
- Accessing radiology podcasts, interviews, and videos (e.g. YouTube, iTunes)
- Social bookmarking (marking and sharing interesting documents, e.g. Pinterest)
- Finding clinical cases and images (e.g. Facebook quizzes)
- Sharing or viewing slide shows containing relevant information or educational material (e.g. SlideShare)

Radiological professional organisations and scientific societies are increasingly maintaining profiles on SoMe, mainly Facebook and Twitter (Table 6) [23–25]. The ESR uses SoMe to communicate with its members and with people interested in the organisation, to facilitate communication among members, to promote both the ESR and ECR (including their services and projects), and to provide an additional service channel. The purpose of the ESR social media channels is to connect with radiologists and members on a global scale. The ESR monitors and evaluates when, where, and how the current channels are being used in order to further optimize its services. Table 7 provides an overview of the main ESR channels.

**Table 6:** Radiological societies and social media

	myESR	RSNA
Number of Facebook fans	157,000	50,000
Number of Twitter followers	4,830	15,000

**Table 7:** The main ESR social media channels

Social network	Name	Number of fans
Facebook	myESR page	157,000
	ESR Rising Stars page	3030
	European Radiology page	7900
	Insights into Imaging page	2500
	ESOR (European School of Radiology) page	2100
	European Diploma in Radiology (EDiR) page	4100
	International Day of Radiology page	12,700
Google	myESR Google page	73
YouTube	myESR YouTube channel	615 subscribers
Twitter	myESR Twitter	4830 followers
Blog	myESR Blog	7700 monthly visits
Pinterest	myESR Pinterest	207 followers

## Popular social media for radiology

In this section we aim to provide an overview of some of the more common and popular SoMe platforms that can be used in radiology to exchange information and interact with the online community, professionally or privately. We provide more detailed information on those social media platforms providing relevant and interesting information for radiologists [26].

### LinkedIn

LinkedIn is a professional peer-to-peer network that enables its users to search for expertise across the social network. A user can invite other LinkedIn users to join his/her network. In contrast to Facebook, LinkedIn relies more on professional relationships than on direct “friendships”. It can be considered an online networking service that focuses on connecting users for professional reasons and for purposes of soliciting and recruitment. Although it provides access to several “groups” that are related to radiology, the degree of social interaction among its users and its educational value remain rather limited compared to other SoMe platforms.

### Facebook

Facebook (www.facebook.com) is the most popular platform today among the general public, including physicians. In general, anyone who has been added to a list of Facebook “friends” is able to view all the material published by their friends, and even posts made by people outside their own network but associated with friends. The Facebook privacy settings currently allow each user to define more precisely who is able to see or read the messages posted by that user. Educational opportunities related to SoMe and networking are progressively migrating to sites such as Facebook. On the myESR Facebook page (http://www.facebookm/myesr), a wide array of topics are addressed: news related to radiology and medical science, news regarding the European Society of Radiology’s annual meeting (ECR) and about the society itself, and referrals to other ESR channels. The easiest way to find radiology-related information on Facebook is by typing “radiology” in the search bar at the top of the Facebook page. In addition to several radiological organisations and scientific journals, many educational pages can be found on Facebook, including Radiopaedia (https://www.facebook.com/Radiopaedia.org), Radiology Signs (https://www.facebook.com/RadiologySigns), and CTisus (https://www.facebook.com/ctisus). Cases on Facebook are often presented in contest format, such as the Society of Abdominal Radiology’s "Gettable Case of the Week" (https://www.facebook.com/SocietyOfAbdominalRadiology), Radiopaedia.org’s daily cases, and the weekly cases of the BIDMC [Beth Israel Deaconess Medical Center] Division of Body MRI (https://www.facebook.com/pages/Division-of-Body-MRI-at-BIDMC/1533647120214439). In May 2015, the three most popular educational Facebook pages based upon the number of “likes” were Radiology Signs (more than 753,000), LearningRadiology (more than 728,000), and Radiopaedia (more than 339,000). Facebook users can collect their favourite radiology pages by creating an “interest list” on their own Facebook page. In such an interest list (e.g. “radiology education”), all posts from the pages they have added are automatically displayed, giving them a structured overview of all relevant radiology information.

### Twitter and microblogging

**Fig. 3:**
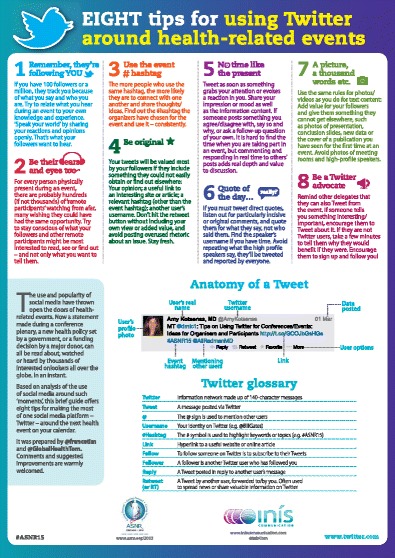
Flyer with tips for using social media during the ASNR 2015 meeting. From: personal communication with Amy Kotsenas during ASNR 2015 meeting, courtesy of Tim France and his team at Inis Communication [26]

Microblogs are SoMe platforms with limits on the amount of content that can be included in a single post, meaning users must be more creative in choosing the information they share and how they share it [4]. Twitter (http://www.twitter.com) is the most prominent example of microblogging. Its users can send and read messages up to 140 characters, which are direct and short communications that may contain links to pictures, documents, websites, or videos. In tweets (Twitter messages), the hash character (or number sign) is often used in front of a word, which makes it easier for users to find messages with a specific theme or content. For example, entering #ECR2015 allows users to find all messages that have been tagged with the label ECR2015. The “hashtag” refers to the combination of the hash character and a tag or label. Twitter is a rather asymmetrical medium: users who subscribe to someone’s “feeds” (postings) are not automatically considered a peer of the tweeter (sender). The subscriber is shown what the sender posts in a news feed style format, but the relationship is not reciprocal, meaning that the subscriber’s tweets will not automatically appear in the sender’s Twitter list. Twitter currently has more than 302 million monthly active users; about 500 million Tweets are sent per day. Radiologists using this medium can follow radiological journals, organisations, and other “tweeting” colleagues. By choosing a good mix of these sources, especially those that tweet links to high-yield content, radiologists are able to create an individually tailored and constantly updated curated source of medical information. Some radiologists and nuclear physicians are using Twitter to post interesting cases with anonymised medical images. Enthusiast followers like to browse through these short cases and comment on them. Some examples include the Radiopaedia.org account (founded by Frank Gaillard) (https://twitter.com/radiopaedia), CTisus.com from Elliot K. Fishman (https://twitter.com/ctisus), LearningRadiology from William Herring (https://twitter.com/radsigns), and SwissNuclearDoctors from Gaël Amzalag (https://twitter.com/NuclearDoctors). Microblogging platforms in addition to Twitter are available as well, such as FriendFeed (http://blog.friendfeed.com) and Tumblr (https://www.tumblr.com). All these platforms feature medical pages, but most users focusing on medicine and healthcare prefer to use Twitter [1]. The myESR Twitter account (https://twitter.com/myesr) has approximately 4,800 followers, who are automatically informed about the latest news and information from the ESR. The account is busiest and sees its peak interaction during the annual ECR meeting. Topics addressed on Twitter during the meeting are similar to those on Facebook, but the communication is quicker and more highly interactive. We consider it useful to distribute a digital Twitter manual to attendees during scientific meetings in order to make the most of the online social platform. Such a guide was provided at the 2015 ASNR meeting in Chicago. In addition to tips for using Twitter during the event, the anatomy of a tweet is explained, as shown in Fig. 3. At the Tweet Chat session of the ASNR meeting, valuable advice was provided for social media novices, such as remembering to use the “meeting hashtag” in each tweet (e.g. #ASNR15), which helps other users find messages relating to this meeting. It was also suggested that when quoting another Twitter user, the tweet should include their “Twitter handle”, which is the Twitter username of the quoted person preceded by the @ sign (e.g. @johndoe). Including a picture from the event in a tweet is most valued by followers unable to attend the meeting [17, 27].

**Fig. 4:**
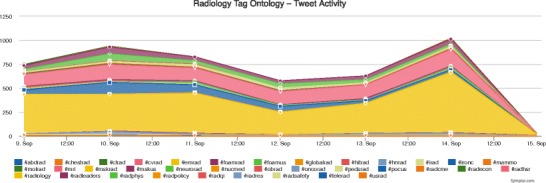
Radiology Tag Ontology displaying the activity on Twitter related to radiology. Each hashtag is represented by a different colour, e.g. #abdrad (abdominal radiology) is dark blue. Source: http://bit.ly/1I9wD75. Accessed Sept 2015

The “Healthcare Hashtag Project” (http://bit.ly/1ds8Iiy) is a website where all health-related topics discussed on Twitter can be found, including the "top-trending” subjects, diseases, and conferences. By typing “radiology” in the search field, the hashtags and topics related to radiology most often used on Twitter are displayed, including all information related to radiology meetings discussed on Twitter. On the right side of the page, the top radiology influencers are displayed. Influencers are usually ranked based upon the number of tweets, number of mentions received, and number of impressions created. By clicking on the “Conferences” tab, all healthcare conferences for each year are displayed. Clicking on the name of each conference or its corresponding hashtag will display an overview of Twitter activity related to that meeting. For example, when searching for or choosing #ECR2015, an overview of the meeting is displayed, along with analysis of the Twitter activity during the event, a list of the most influential tweeters by activity and following, and the latest tweets using hashtags related to ECR 2015 (http://bit.ly/1JbgIF4). The “Radiology Hashtag Ontology” project is an effort to more efficiently organise radiology-related content from social media, with the goal of enabling as many users as possible to meaningfully contribute to conversations on imaging-related healthcare (http://bit.ly/1I9wD75). A standard lexicon for radiology tweets has thus been proposed in order to make tweets more traceable and usable for radiologists. Users are also invited to propose new hashtags themselves. On the same website, all Twitter activity related to radiology is monitored, including the total number of participants. In Fig. 4, the Twitter activity is displayed for each hashtag related to radiology. In Table 8, some advantages and reasons for radiologists to use Twitter are summarised.

**Table 8:** Reasons for radiologists to use Twitter

- To get a quick overview of news and literature by following people with similar interests, educational leaders (“radiology influencers”), scientific journals, and professional (radiology) organisations	
- To engage in discussions and chats with peers, without hierarchy	
- To gather radiology-related information and news	
- To participate in Tweet Chats during radiology meetings	
- To become a curator of information, to inform colleagues where you stand on topics	
- To propagate expert opinions and research findings	
- To increase the visibility of radiology, both for patients and medical professionals	
- To engage as a medical imaging expert in dedicated professional SoMe platforms	

### Instagram

Instagram (https://www.instagram.com) is a social networking service designed for online mobile sharing of pictures and videos. The service was launched in 2010 and acquired by Facebook in 2012. In March 2015, the company had a total of more than 300 million active accounts. The well-known radiological teaching site Radiopaedia can also be found on Instagram, with a collection of interesting cases and videos, including their online discussions (https://instagram.com/radiopaedia). With 13,000 followers, it is by far the most popular radiology source on Instagram. The American College of Radiology (ACR), which posts pictures of ACR-related events or entertaining pictures (https://instagram.com/radiologyacr/), has more than 1,400 Instagram fans. Radiology_whisperer (https://instagram.com/radiology_whisperer/) is a Chicago-based radiologist posting interesting radiology cases on Instagram, with 1,681 followers.

### “Figure1”

**Fig. 5:**
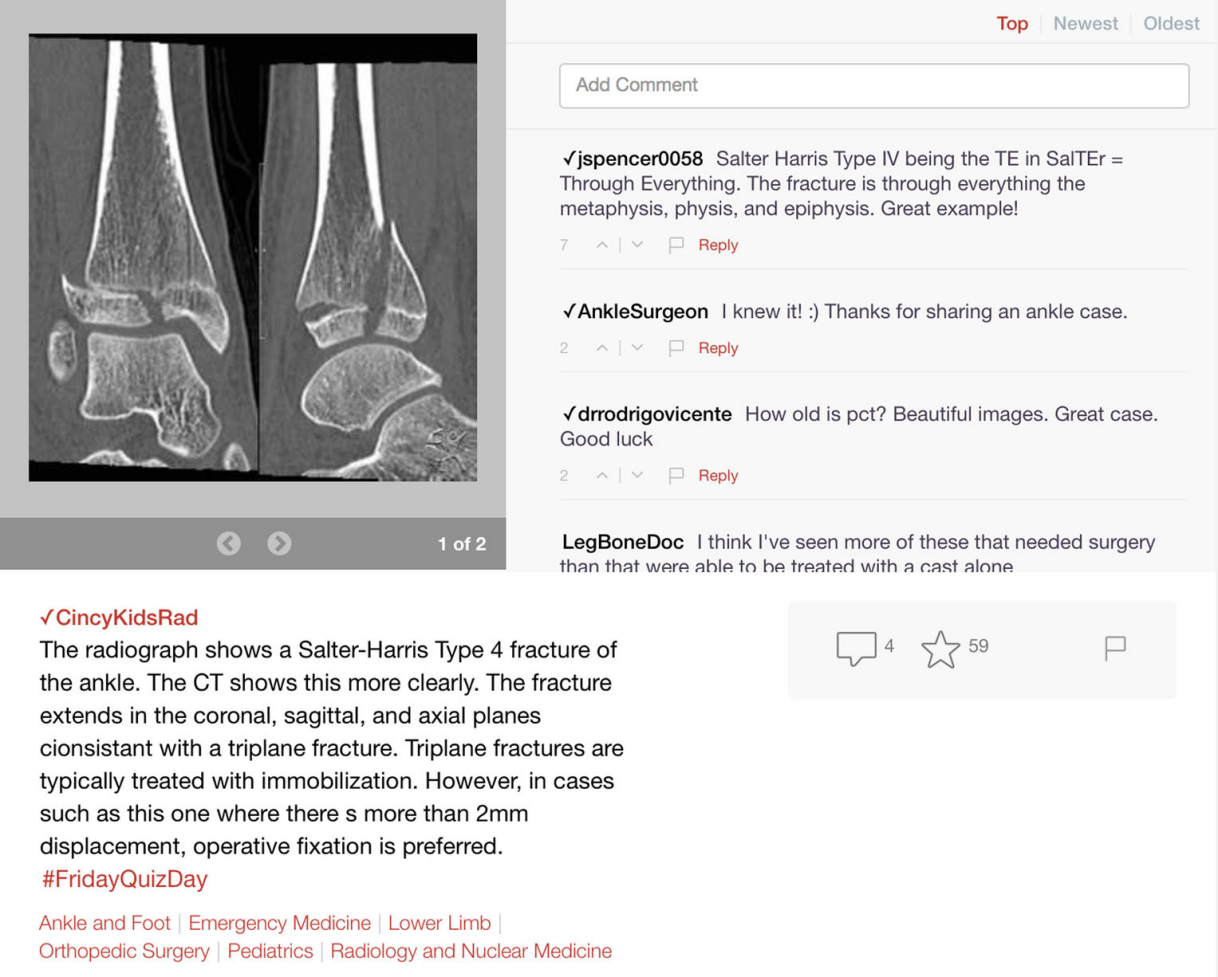
Example of a radiological image posted on Figure1.com, which was discussed by several orthopaedic surgeons and a nurse. In this case, no radiologists are involved. Source: http://bit.ly/1K27JFh, last accessed May 15, 2015

The Figure1 mobile app has been touted as “Instagram for doctors” (https://figure1.com) [28, 29]. This crowd-sourced medical library enables physicians and medical residents to post and discuss clinical images from their mobile devices and to digitally archive interesting cases. The main goal is to facilitate and support social networking among young doctors-in-training when they are confronted with complex or interesting cases. The concept is based upon the idea that “images are a great way to teach and tell a story”. In contrast to Instagram, confidentiality and patient privacy are well protected. Several tools have been incorporated to automatically remove sensitive patient details. Faces are automatically obscured: body tattoos can be manually blocked, and a human privacy moderation team reviews each photo or video before it is added to the database. Healthcare professionals willing to participate are required to sign up and undergo a validation procedure before they can actively contribute to the platform. The app has been launched in several English-speaking countries and has gained approval from the health authorities in several other countries including Germany, France, and the Netherlands. The platform is unique in the sense that its users can receive assistance in interpreting difficult cases from a “collective brain” of healthcare professionals. Furthermore, the extensive database is easily searchable for information on particular body areas and related diseases. In this platform, radiologists are able to contribute actively by participating in discussions in which radiological images are posted, or by posting interesting radiology cases for training and teaching purposes. An example of a Figure1 discussion about a radiological examination is shown in Fig. 5. In our opinion, this medium is a good example of a professional social platform with the potential to increase cross-border medical collaboration and teaching. However, the information posted is not systematically peer-reviewed, which is considered by some as a limitation or potential risk to using this type of educational platform compared with the more traditional media currently being used for publication of scientific and educational material.

### Blogs

Blogs are the most traditional of social platforms [4]. On a blog website, people can convey their thoughts, ideas, and stories. The posts are typically displayed in reverse chronological order (most recent post comes first). Radiologists can use such platforms to express a personal opinion or commentary for which they would never use an official or scientific channel. Several websites can be used for creating personal websites and writing blogs, including Blogger.com (https://www.blogger.com), WordPress (https://en.wordpress.com), and about.me (https://about.me). Several examples of radiology blogs can be found in Table 9.

**Table 9:** Examples of Radiology blogs

Name	Author(s)	Link
Sumer’s Radiology Blog	Sumer Sethi	http://sumerdoc.blogspot.com
Radiopaedia blog	Frank Gaillard and guest bloggers	http://radiopaedia.org/blog
Diagnostic imaging blog	Guest bloggers	http://diagnosticimaging.com/blog
UCSF Radiology blog	UCSF Department of Radiology and Biomedical Imaging	http://blog.radiology.ucsf.edu
radRounds blog posts	radRounds Radiology Network team, guest bloggers	http://radrounds.com/profiles/blog/list
ESR blog	Staff and members of ESR	http://blog.myesr.org/posts/
Musculoskeletal radiology	Keshav Kulkarni	http://musculoskeletal-radiology.blogspot.be/
medGadget	Editorial team	http://medgadget.com/radiology

### YouTube

YouTube is a platform created for publishing videos, and also contains numerous "social" features for sharing and viewing a variety of media including lectures, case studies, presentations, trailers, teasers, video reports, and interviews. Radiology organisations and radiologists also use YouTube for education and information. The myESR YouTube channel (http://bit.ly/1ACwRRi) had more than 122,000 views in May 2015, including 615 subscribers [30]. The Radiology Channel (http://bit.ly/1FMeWpY), which is part of the Radiopaedia.org platform, provides videos with radiological cases and courses.

### SlideShare

SlideShare (http://www.slideshare.net) is a hosting service where users can upload presentations and other documents (infographics, documents, videos, PDFs, webinars), which can be viewed publicly or privately. It now comprises more than 15 million uploads from individuals and organisations on a variety of topics, including health and education. Presentations can be discussed online or distributed through other SoMe. In the fourth quarter of 2013, the site averaged 60 million unique visitors per month. It is currently ranked among the world’s top 10 tools for education and learning. A simple search for “radiology” results in more than 330,000 relevant presentations. As mentioned earlier, the fact that none of the information available on this platform is peer-reviewed could be considered a potential risk concerning the quality and educational value of this medium.

### Scientific networks

Researchers and scientists have several online social networks at their disposal, including ResearchGate, Mendeley and Academia.edu. Every user of these platforms is able to create his/her own scientific profile and to share this information with others. ResearchGate.net (http://www.researchgate.net/) is the most popular platform, currently with over five million members, of whom more than 600,000 work in a medical-related field [31]. This platform allows its users to generate a free digital object identifier (DOI) for research that they add to their profile, making their work, conference papers, and posters more easily traceable and citable. For each user an “RG Score” is automatically published on his/her profile as a measure of the user’s scientific reputation. The score is calculated based upon all of his or her publications and the way that the research is received by peers. In this respect, ResearchGate is approaching the more traditional peer review process, since the scientific quality of information provided is more or less guaranteed by the RG score of the author. This is in contrast with most other social media platforms, where the acceptance of published data is based rather upon an “open review” system. Mendeley (https://www.mendeley.com/) is a free reference manager and academic social network that enables its users to make their own fully searchable library and to share it with other researchers and students. Academia.edu (https://www.academia.edu/) is a platform for academics to share research papers, and attracts more than 36 million unique visitor each month. Some of the advantages of online research networks are summarized in Table 10.

**Table 10:** Advantages of online scientific networks

• Opportunity to find collaborators from around the globe	
• Possibility of joining, creating, or participating in research-specific areas	
• Easy access to numerous scientific publications and abstracts	
• Facilitation of online scientific communication and peer-to-peer learning	
• Access to jobs in science, networking opportunities in the research arena	

## What is the value of traditional scientific media compared to SoMe?

For the last 150 years, the sharing of information within the medical community has largely occurred through presentation of work in scientific meetings and publication in scientific journals. Abstracts submitted for presentations must be scrutinised by reviewers and accepted before they can be presented. Similarly, in scientific journals, the articles submitted undergo a rigorous (blinded) reviewing process, thus ensuring, to the greatest extent possible, that the information is correct and reproducible. While far from perfect, this process does have a positive effect on the quality of the presented or published material. Medical journals are ranked according to the impact they have on the scientific community. Calculation of the “impact factor” of a journal is based upon the number of citations divided by the number of published articles in that particular journal for any given year. In other words, with the “traditional” publication method, a more hierarchical pathway must be followed before the information is distributed. Papers published in journals with a high impact factor do have more “weight” and influence in the medical community than those published in journals with a low impact factor or none at all. Similarly, there are also high-profile and low-profile meetings. In SoMe, on the other hand, there are no barriers or hierarchy, and there is no peer review process. Anyone can publish anything online, unhindered by a lack of in-depth knowledge on a specific topic. Although this can be perceived as a disadvantage or potential risk in using SoMe to obtain medical information, SoMe should not be regarded as a completely uncontrolled information resource. Since there are no hierarchical or editorial barriers, users of SoMe can freely post their critiques and comments. In this respect, an “open review process” among the virtual community in SoMe replaces the more traditional review procedure; in other words, so-called collective intelligence is used to accept or reject the posted information. This open review process or assessment by the online professional community may evolve into a new type of scoring system resulting in another form of stratification of more and less relevant information when implemented on a large scale in SoMe. Such a scoring system is already being applied in ResearchGate, as explained in the previous section. SoMe can also have a significant impact on the visibility and popularity of material published in scientific journals. Several radiological organisations and journals are exploring how they can use SoMe to extend the reach of the traditional Web-based or printed publications. A recent study provided evidence that dissemination and discussion of scientific information via a radiology blog and SoMe can significantly increase the number of readers in comparison with more traditional publication venues [32]. This study concluded that there is growing awareness among researchers and academic and professional society leadership that further integration of SoMe within their communication strategies must be seriously considered, since it might also lead to more extensive networking among people with common interests and priorities.

## Conclusions

Radiologists can use online social networking not only to enhance their communication and collaboration with peers, but also to retrieve relevant information that can help in decision-making, for quick access to educational sources, and to facilitate communication and the exchange of information used in research. Physicians, including radiologists, should be aware of these new developments and their advantages, but also must understand the potential disadvantages, pitfalls, and limitations. Although SoMe platforms hold great potential and have a bright future, recommendations and guidelines must be provided by policymakers and professional organisations with the primary intention of keeping the use of social media in balance with professional values and ethics [23, 31]. In our opinion, the development of more SoMe applications for dedicated professional use is needed to allow the effective, efficient, and secure exchange of medical imaging information within the social space and to facilitate collaboration between radiologists and clinicians at a local, national, and international level. We would encourage radiologists to explore the world of social networking with this background knowledge in mind, and to evaluate ways to optimally integrate these tools into their radiology practice. For this purpose, we have included below a list of Dos and Don’ts that can be used by radiologists as an aid in exploring the virtual world of social media, safely and securely (see Table 11).

**Table 11:** Dos and Don’ts for radiologists using social media

Dos	Don’ts
• Be selective in who you add to your list of friends, keep it restricted to people you know and respect	• Don’t make the mistake of assuming that social media can replace “serious” scientific publications, but consider social media as a useful additional source of information
• Protect your reputation as a medical professional	• Don’t share confidential information about patients
• Promote your work towards professionals, colleagues, and friends	• Don’t send negative reactions
• Think positively in your reactions, do not insult people	• Don’t believe everything you read
• Read and check your messages before you share or re-tweet information (you are what you tweet and share)	• Avoid having multiple professional profiles, and protect your credibility
• Be sure about any content you may want to share	• Don’t spread yourself too thin; start with one, or at most two, social networks, and go from there
• Be wary of spam, and don’t send spam yourself	• Don’t abuse hashtags
• Be focused and develop a direction for sharing information	• Don’t become obsessed about the number of followers
• Share without any ulterior motives	
